# Supplementary material for: HippoUnit: A software tool for the automated testing and systematic comparison of detailed models of hippocampal neurons based on electrophysiological data
Source: PLoS Comput Biol. 2021 Jan 29;17(1):e1008114. doi: 10.1371/journal.pcbi.1008114 (PMC7875359; doi:10.1371/journal.pcbi.1008114)
Supplement: Attachment — Submitted filename: PLoS Comp Bio Review.pdf [file pcbi.1008114.s002.pdf]

The manuscript of Saray et. al. describes a rigorous and impressive platform, HippoUnit, for the testing of neuron models in a quantitative fashion against a uniform set of experimental data. The technical accomplishment alone is quite impressive, as neuron models are often coded in such distinct fashions that creating a platform that can be readily applied to a wide range of such models is a significant challenge. Tools to perform such tests in a widely applicable manner are, indeed, much needed. On this alone, this manuscript merits strong consideration for publication at *PLoS Computational Biology*.

While I am extremely impressed by the platform (although there are issues with it that need to be addressed, discussed as "major comments" below), I am concerned about the way it is presented by the manuscript. The authors assert throughout that there is a need for "community models" and models that account for a large number of diverse neural dynamics. As currently written, the text seems to argue that this is a clear consensus within the computational neuroscience community and that the inability of models to meet these standards is a clear failing, in turn motivating the tool presented in the manuscript. However, this question is still very much open for debate: many computational neuroscientists have highlighted how "cell-to-cell variability" (see Golowash et. al. *Journal of Neurophysiology* 2002 and Ransdell et. al. *JNeurosci* 2013) necessitates the creation of a multitude of neuron models even from the same population (see Marder and Taylor *Nature Neuroscience* 2011 and Marder and Goaillard *Nature Reviews Neuroscience* 2006, as well as the numerous citations in both pieces on the inherent biological variability in neurons), while others have questioned whether a single "realistic" neuron model is even feasible (see Almog and Korngreen *Journal of Neurophysiology* 2016). This underlies my primary concern with the manuscript: in the context of the "debate" outlined above, the manuscript currently lacks a sufficient argument as to whether the testing of multiple neuron models against an external set of experimental data, yielding a quantitative "score" of its validity, is justified and necessary for the field.

Thus, I feel significant work is required to properly contextualize this manuscript within this ongoing debate in the field, more clearly articulate the "problem" that this platform solves, and craft an argument as to why that problem is a significant concern to the community (and in turn how, in solving it, this manuscript moves the field significantly forward). Independent of my personal opinion on this debate, I remain entirely open to publication of this work as long as a reasonable and clear argument is made on this topic, including an acknowledgement of the alternative opinion (and likely a discussion of how this tool will be useful to those who may not agree with the premise that the search for more "uniform" models is necessary). Addressing the following concerns would likely prove useful in crafting this aspect of a revised manuscript:

- How would the pursuit of a "community model" fit with the known "cell-to-cell variability" as described in the articles (included above) by Marder, and her (and others') conclusion that this necessitates the creation of multiple models to reflect this diversity?
- Why is the fact that there are "a large number of different models of the same cell type… that were developed for different purposes" necessarily a negative, as is implied in the text at Line 80? Put alternatively, on Line 690 the authors acknowledge that "the behavior of the different models is very diverse". Is this a "feature" of the actual biological system, or a "bug" in the current modeling state of the art? It seems that the

paper is arguing the later, although many (see the citations included here) would argue the former, or that this diversity is simply a necessity given the current state of the art.

- The analogy to software development (Line 87) is an interesting one but lacks sufficient justification for its application here. Why is this analogy appropriate?
- On Line 92 the authors admit that the pursuit of "community models… has rarely been attempted in neuroscience." This begs the question of *why* this is the case. If this pursuit has rarely been attempted in the decades that neuron models have been in use, might there be justification for this? If so, what would be the counterargument to this justification that motivates the pursuit outlined in this paper?
- The choice of the "features" used to judge the models, as well as the experimental data to compare to, seems in itself inherently subjective, and counter to the idea that HippoUnit provides an "objective" measure of a model's accuracy. Why were these specific features, and the corresponding experimental data, chosen? Why are these features enough to capture the entirety of a neuron's dynamics?
- What about the impact of neural morphology? The authors themselves discuss the variety of morphologies amongst the models that they test, and it is well established that this variability impacts neural dynamics (see Mohan et. al. *Cerebral Cortex* 2015 and Eyal et. al. *eLife* 2016 as just two examples). Is it appropriate to compare dynamics from different neurons, that necessarily have different morphologies?

While I feel addressing these concerns is necessary as the paper is currently constructed, I acknowledge that this would veer somewhat into the realm of a "philosophical" argument (see Almog and Korngreen) that may not be appropriate or reasonable in a "Research Article". However, a straightforward solution would be for this piece to be reworked as a "Methods" article. As I've mentioned above, the technical prowess required to implement HippoUnit is impressive, and the authors have done a good job outlining multiple fashions in which this tool can be used effectively. Rather than making an argument about the necessity of "model validation" and "community models" that this software may not be equipped to fully answer, a simple presentation of the unique tools provided by this platform would facilitate the use of this tool by computational neuroscientists regardless of their opinion on the "debate" I've described here in detail. There are already many portions of the paper that read like a "Methods" article, so I feel that making this change would not be an onerous task.

Regardless of whether the authors choose to address these concerns via a more significant revision and resubmission as a "Research Article", or by reframing the manuscript as a "Methods" piece, I remain open to reevaluating a piece that addresses the concerns I've outlined (and quite interested and excited to see how the authors choose to address these questions). In summary, while HippoUnit itself is impressive and should be of great use to the computational neuroscience community, I feel the manuscript as written may be "overreaching", and in so doing requiring that the piece answers "philosophical" questions that this tool may not be suited for. However, I believe that with some alteration to its presentation, the manuscript could "describe outstanding methods of exceptional importance that have been shown, or have the promise to provide new biological insights" (taken from the "Scope" for a Methods article), and thus merits strong consideration under the Methods banner.

Some additional comments, both major and minor, that should be addressed in revisions are included below:

Major:

1. As an important example of the issues caused by the lack of proper contextualization, consider the following sentence beginning on line 56: “Applying our tests to several models available in the literature, we show that each model is able to capture some of the important properties of the real neuron but performs badly in other domains.” A similar statement is made on Line 743: “From this figure it is even more clearly visible that each model fits some experimental features well but does not capture others.” This is not necessarily a surprising or novel finding. While to some this reveals the limitations of computational modeling, others see this simply as the reality of the field given the current “state-of-the-art” (the arguments of Almog and Korngreen are illustrative of this perspective). Addressing this concern in the overall context of the current state of neuronal modeling would be an ideal place to start in crafting the “argument” for “community models” that I think is necessary in this piece.
2. The last line of the abstract, stating that this framework has “the aim of facilitating more reproducible and transparent community model building”, is a fantastic statement of the utility of this tool. I feel that this could serve as an extremely useful “thesis statement” for a Methods type piece, as it completely sidesteps the debate about the need for “community models”. If the authors choose to keep this as a Research Article, this would need to be expanded upon, as more concrete examples of how this has been accomplished using this tool would be necessary.
3. A discussion of “cell-to-cell variability” (see Golowash and the articles by Marder) must be included as a “counterpoint” to the second paragraph of the introduction beginning on Line 74. Some neuroscientists would argue it is inappropriate to constrain or compare a neuron model with data from other cells, let alone from other animals and other experimental conditions. This issue arises throughout the paper, as the platform compares cell models to results taken from other literature, and necessarily other cells/animals/experimental protocols. This needs to be directly and thoroughly addressed. This issue comes up again in the Results at Line 766, amongst other places.
4. Evaluating models based on a “common standardized criteria” (Line 97) might be useful, but the argument for this needs to be more fleshed out. Furthermore, determining *what* these criteria are itself is a complicated and potentially contentious endeavor, so how these criteria were chosen by the authors needs to be more thoroughly developed.
5. I love the sentence beginning on Line 118: “The test suite that we have developed allows for the quantitative comparison of the behavior of anatomically and biophysically detailed models of CA1 pyramidal neurons with experimental data in all of these domains.” This feels like another part of an ideal “thesis statement” for a Methods piece: instead of having to make the argument for the “significance” of the results obtained using this tool, the authors instead need only describe its power and utility.
6. The way HippoUnit is used in the *development* of new models seems to be one of the most exciting features of this tool, yet it is treated almost as an “afterthought” in the manuscript (it isn’t even mentioned in the introduction until Line 123, the final

paragraph). Were this paper to be recontextualized as a Methods article, more strongly emphasizing this application of HippoUnit may significantly benefit the piece.

7. The paragraph beginning on Line 207 is a bit concerning. The statement that the voltage responses "can strongly depend on the experimental method" is true, and arguably could be seen as a flaw in the methodology of HippoUnit. Addressing this by saying the features were "extracted from two different datasets" does not seem sufficient to address this concern, especially considering the known biological variability between similarly classified cells (again, see the various citations above). Similarly, the argument about how this data was determined to be "typical" needs expansion. Many would argue that comparing models to "typical" values is itself a problematic methodology given cell-to-cell variability (see the various citations above); thus, this potential criticism needs to be directly addressed and the choices made in the implementation of HippoUnit more fully justified.
8. HippoUnit is described as being applicable generally to a wide variety of neuron models, implemented in a variety of fashions: the authors assert in the Results that (Line 591) "HippoUnit can be run on neural models that are built in the NEURON simulator software, without any further coding required from the user." However, in the Methods (Line 449) the authors state that in order to test one of the models described in the paper, an extra modification had to be made to synapse functions. Why is this not evidence that HippoUnit is not as "generalizable" as advertised? Why is this not included in the HippoUnit code proper, but only in a secondary location? The paragraph in the discussion beginning on Line 1330, in which it is revealed that significant additional work was required to implement the models studied in this work in HippoUnit, also seems contradictory with this, and thus needs to be disclosed more clearly and conspicuously.
9. The issue of comparing different neurons with different morphologies (described above) should be discussed in more detail regardless of whether this piece remains a "Research Article" or becomes a "Methods" article.
10. The issue described in the paragraph beginning on Line 776, in particular that "the models are not compared to exactly the same set of features in the two cases" and that this influences the error scores, could be interpreted as a flaw in HippoUnit that could be addressed by further development of the underlying platform. If it is not, some elaboration on this issue would be useful in assuaging any further criticisms, as the solutions described in the following paragraph seem "ad hoc" at the moment.
11. On Line 845 the authors discuss how three models were able to "cheat" the Depolarization Block Test. This could be read as undermining the overall efficacy of HippoUnit, especially given its stated goal to be applicable to a wide variety of models. At minimum, an explanation as to why further steps weren't taken to adjust the platform for these "cheats" is necessary (a statement that this would be computationally problematic/inefficient/etc. would be completely reasonable if this was the case), as well as perhaps further discussion as to how one might deal with similar "cheats" when this platform is applied to other models.
12. The fact that the Golding et. al. models do not perform well on the Back-Propagating Action Potential Test, despite the fact that they were developed to account for this feature (Line 1109), is critical. If this model was designed to exhibit this behavior, what

does that say about the test implemented in HippoUnit? One interpretation might be that this shows why it might be inappropriate to compare models to experimental data obtained from different neurons in a different experimental setting. Something similar appears to be the case for the Gomez Gonzalez et. al. models and the Oblique Integration Test. Both these points need to be thoroughly addressed.

13. The opening sentence of the paragraph beginning on Line 1303 is very well put. However, it seems out of place relative to the rest of the paper, which seems (intentionally or unintentionally) to advocate that models that aren't validated in a wide range of settings aren't "good enough". I think the rest of the paper could benefit from keeping the tone/perspective from this section (which may be facilitated by the transition to a "Methods" article). In particular, stating that "no single model should be expected to achieve an arbitrarily low score on all of the validation tests" (Line 1318) is an important statement that needs to be made more prominently in the manuscript.

Minor:

1. On Line 21 the authors state that neuronal models "can be" useful. This seems unnecessarily deferential, especially considering that it is argued how important they are throughout the rest of the paper.
2. The term "validation" is used throughout the paper, but this is a term that can be interpreted in a variety of different ways. I would avoid using this term in the abstract and title if at all possible, and then introduce how this term is being used in this manuscript thoroughly in the introduction. "Accurate" is another such term that might benefit from expounding upon.
3. On Line 179, the assertion that HippoUnit's scores "better fit its tests and the observations belonging to them" needs to be elaborated on further… "better" is an inherently subjective term and requires some sort of argument justifying its use.
4. The fact that the AP amplitude has such a broad range (Line 236) could be interpreted as a strong piece of evidence for a "counter-argument" against the methodology taken in designing HIppoUnit. A stronger argument as to why this is acceptable in the design of this tool is necessary, especially given the context of the "community model" debate.
5. The tool for classifying the apical sections of pyramidal cells (Line 485) is very impressive, and in itself extremely useful as a uniform mechanism for classifying sections in a NEURON model. Might this be more strongly highlighted (especially if the article becomes a "Methods" piece)?
6. In the "Models from literature" section of the Methods (Line 521), each model is described in detail, including what the model was initially designed for. I feel that this knowledge is crucial in order to evaluate the Results, and thus might be more appropriate as the first section of the Results. If the authors choose not to make this change, a brief "refresher" on this topic would be useful to begin the Results.
7. The sentence beginning on Line 568 regarding the modeler's feedback seems superfluous, especially since feedback was received from only two of the groups. I would suggest eliminating this sentence, or if not further expanding upon what this feedback contributed to the work.

8. The opening sentence of the Results (beginning on Line 577) already reads more like a “Methods” paper than a “Research Article” (and is extremely well written!), and might facilitate that transition if the authors choose.
9. The argument made in the paragraph beginning on Line 661 is already made in the Methods (i.e. that the data used in HippoUnit matches the available literature), and probably doesn’t need to be made in full again.
10. It may be illustrative for the authors to compare their Figure 3 to some of the figures in the two Marder papers cited here. In those papers, very similar types of figures are used to argue that biological variability exists in neurons and necessarily should influence the creation of neuron models, whereas here the authors seem to use this figure as evidence that the existing neuron models are lacking. Reconciling this could prove useful in crafting the proper context for this manuscript.
11. If “not all the observation features can be evaluated for each of the models” (Line 819), is it appropriate to include those features in HippoUnit? Why not eliminate them and only compare “apples to apples”, i.e. features that can be tested in all of the models? This should be addressed in more detail.
12. I would suggest reworking the opening paragraph of the Discussion (beginning on Line 1264). Many of the statements in this paragraph are opinions (albeit well supported ones), but as currently written they come across as statement of fact. Some more citations and specific examples backing up these statements would likely help.
13. A mention of the Neurodata Without Borders initiative seems wanting in the paragraph beginning on Line 1346.
