# Supplementary material for: HippoUnit: A software tool for the automated testing and systematic comparison of detailed models of hippocampal neurons based on electrophysiological data
Source: PLoS Comput Biol. 2021 Jan 29;17(1):e1008114. doi: 10.1371/journal.pcbi.1008114 (PMC7875359; doi:10.1371/journal.pcbi.1008114)
Supplement: Attachment — Submitted filename: response_to_reviewers_Sarayetal2020.docx [file pcbi.1008114.s003.docx]

Dear Prof. Lytton and Prof. Blackwell,

We sincerely thank you and both reviewers for your constructive comments on our manuscript, and for the invitation to submit this substantially revised version.

We decided to follow your recommendation to convert the present article into a pure Methods paper. We removed much of the original text that argued for the necessity of developing automated test suites from the angle of community model development, and added several more broadly applicable arguments for the utility of our software tool. This required a substantial revision of the manuscript, especially in the Introduction and Discussion, but also in other sections. We have also carefully considered all other (major and minor) comments from both reviewers, and revised all parts of the manuscript accordingly. More specifically, in addition to appropriate changes in the main text, all figures were updated, the figure captions were revised and extended, and a new table describing key electrophysiological parameters was added. In the uploaded "Related Manuscript" file we marked the changes of the manuscript in yellow, and to make it easier to read and follow, we still included the figures and the supporting information in this version of the revised manuscript. Please find our point-by-point response to all comments of the reviewers below.

We also thank you for the suggestion that we could write and submit an additional perspective article on the more general, philosophical and practical issues regarding consensus and community models. We agree that this is an interesting and probably controversial topic that would be worth exploring (possibly from multiple viewpoints), but we will need more time to decide whether and how we wish to pursue this opportunity.

We hope that our revised submission will be received favorably at the journal, and look forward to hearing from you.

Yours sincerely,

Sára Sáray

Responses to Reviewers:

Reviewer #1: General Comments

* The authors have developed a very interesting, useful tool which promises to greatly improve the development of biophysically detailed neuron models, and I hope that tool will go on to be widely used in the field. The data presented here provide a good demonstration of capabilities of HippoUnit and how it can be used to improve model development.

We thank Reviewer #1 for his/her positive assessment of our work, and especially for the constructive comments that allowed us to further improve our manuscript.

* While the authors convincingly demonstrate the utility of their tool, the manuscript says little in the way of what it can teach us. Though the authors acknowledge there may be no single model which performs arbitrarily well on all tests, are any of the tests or optimization methods used to create these models more or less important for making them more generalizable? For instance, is their any clear reason the model from Katz et al. 2009 seems to do reasonably well on most tests, while others have more varied performance?

First, we note that the primary purpose of HippoUnit is the objective, quantitative comparison of model behavior with experimental data in specific paradigms. Therefore, simply applying the tool to different models does not automatically provide answers to questions concerning the mechanisms that underlie the models’ behavior. However, more creative applications of HippoUnit can certainly support the investigation of these mechanisms. For instance, by re-running the tests of HippoUnit after the removal of certain ion channels from the model, the contributions of these channels to various aspects of physiological behavior can easily be tested and quantified. The issue of generalization could be investigated further by examining whether the scores in one test can be used to predict the scores in other tests. We have now included a section titled “Applications of the HippoUnit test suite” in the Discussion of the paper (starting in line 1335), which describes other possible scenarios in which our tool is expected to be useful. We are also using HippoUnit in several ways in our ongoing research projects. On the other hand, following the advice of Reviewer #2 and the Editors, we have converted the present manuscript into a Methods article, focusing on the description of our software and demonstrating its use, but leaving the investigation of specific scientific questions (and also related philosophical issues) to future papers.

* A general comment on the figures: They include a lot of information fairly compactly, and that is commendable, but they are almost impossible to decipher unless they are viewed in tandem with the main text. Because I think is a tool that should be adopted by the field, and since many readers tend to skim the figures for something of interest before reading the manuscript in toto, it would greatly improve the manuscript and the chances of HippoUnit's wider adoption if the information in the figures were made more self-evident (more detailed captions, subfigure labels, more clearly labeled axes, etc.).

We thank the reviewer for the general and specific comments on the figures. We have thoroughly revised all of our figures to make them more easily readable, and also added more detailed information to the captions.

* Can you say what it is about the Bianchi model that makes it behave correctly in the depolarization block test or how the other models go about producing behavior that "tricks" it?

As we already mentioned, running the tests of HippoUnit does not directly answer questions about mechanisms, and (especially after converting this manuscript into a Methods article) we decided not to pursue further scientific questions in this paper. However, we believe that, by testing various altered versions of the Bianchi model using HippoUnit, one could identify which ingredients of the model are critical in producing the observed behavior. On the other hand, the way in which certain models may be able to “cheat” the test is certainly relevant for the applicability of HippoUnit, and we have added a paragraph discussing this issue to the section “Depolarization Block Test” in Results (at lines 913-921). The main point is that the behavior of these models is so similar to depolarization block that it is hard to distinguish them using automated methods, although a trained researcher can tell them apart by looking at the voltage traces and other outputs of the test. We were also concerned that adding further (ad hoc) modifications to the test so that it produces the correct output in these particular cases might lead to incorrect results in future test cases.

* It would be very informative to provide some explanation of why the tests applied here were chosen (Somatic Features, PSP Attenuation, etc.), as well as what tests could have been included and why they were not.

Two basic considerations drove the selection of tests for implementation in HippoUnit. First, we tried to include tests that characterize different aspects of neuronal electrophysiology, and that collectively provide a strong set of constraints on the input-output function of CA1 pyramidal cells. However, we do not believe that the current set of tests completely characterizes the physiology of these neurons; for instance, it would be useful to expand HippoUnit by adding new tests on the signal integration of the basal or the more distal apical dendrites. Second, we were severely constrained by the availability of experimental data required for precise, quantitative comparison. We had to find papers that describe the experimental and analysis protocol in enough detail to allow its exact replication in the simulations, and that provide enough information (in the text, tables, or sometimes figures) to compute the experimental mean and standard deviation for the relevant features. We note that we will continue to extend HippoUnit after the publication of this paper, and hope to receive contributions from other members of the community. We have also started to expand HippoUnit to include other hippocampal cell types.

We discuss this point in the revised manuscript at lines 569-575 and 1458-1465.

Specific Comments

* Fig 1 - I'm assuming that the orange/purple coloring of the dendrites is meant to be basal/apical respectively, but please specify in figure legend.

The figure caption now contains this information about the dendritic types, and a scale bar is also added to the figure.

* Fig 2 - Would it be possible to place scale bars for the morphologies? Or if they are all on the same scale, please say so in the figure legend.

Scale bars have been added to each morphology.

* Fig 3

** What are the solid lines?

** This is a fairly minimalist caption for such a dense figure. Maybe label each panel based on what the protocol for finding each of these features.

** The y-axis labels are a bit cryptic, need to dig through text to find them.

** In the legend, what is the difference between 's' and 'w' in reference to the Golding models?

** It's hard to distinguish experimental values from Poirazi model's values consider using asterisks instead

of circles.

The caption of the figure was extended to answer all these questions. Experimental data are now represented by a black “X” in every figure, which is easier to distinguish from the colored dots.

* Fig 5 - It's not immediately clear that the final/raw scores referred to are the feature error scores, and that a lower score means a better fit to the data. More consistency in how scores are referred to would improve the clarity of the manuscript.

Thank you for drawing our attention to this inconsistency. We are now using consistent terms throughout the entire revised manuscript. We refer to the discrepancy between the target experimental data (observation) and the models’ behavior (prediction) with respect to a specific feature using the term “feature score” (this definition is also included in the manuscript at lines 192-194). The final output of the tests (which is typically the average of all the feature scores) is called “final score” throughout the manuscript.

* Fig. 11 has similar issues to Fig 3 - minimal caption, not obvious what one is looking at without having read the text, y-axis labels are not very informative. Additionally, it would be nice to indicate on the figure where statistical significance is achieved.

We added a much more detailed caption to this figure and indicated statistical significance.

* HippoUnit - How would one about new experimental data or new tests be integrated into HippoUnit? Is there a tool in the package that walks the user through this, or do they have to create their own JSON files 'by hand'?

The way the JSON files are created depends on the nature and source of the experimental data. For the Oblique Integration and the Depolarization Block tests the data were explicitly provided in the text of the papers, therefore their JSON files were easy to make by hand. In the PSP Attenuation and the Back-propagating Action Potential tests the data from the figures of the papers were digitized into tables, then the feature means and standard deviations were calculated and saved into a JSON file by a script. An example of this script has been uploaded to: https://github.com/sasaray/HippoUnit_demo/tree/master/target_features/Examples_on_creating_JSON_files/Magee2000-PSP_att. For the Somatic Features test the feature means and standard deviations are extracted using BluePyEfe (<https://github.com/BlueBrain/BluePyEfe>) from a number of voltage traces recorded from several different cells. Its output can be converted to stimulus and feature JSON files using this script: <https://github.com/sasaray/HippoUnit_demo/blob/master/target_features/Examples_on_creating_JSON_files/Somatic_Features/convert_new_output_feature_data_for_valid.py>

This is now discussed in the manuscript in the Methods (lines 255-260, 375-379) and in the Discussion (Extensibility of HippoUnit – lines 1474-1483)

* The information provided in Lines 232-250 may be better suited to a table.

In the revised manuscript these data are arranged in Table 1, and only briefly described in lines 243-246.

* Lines 607-610 - I'm not sure where in Methods outlines these useful output

We have included this information in the second paragraph of the section “Implementation of HippoUnit”, lines 147-156.

* Line 998: "they own" -> "their own"

Corrected - thank you for spotting the typo.

* Lines 1066-1068: 'With this synaptic weight the first dendritic spike, and therefore the jump in the “measured EPSP vs expected EPSP” curve to the supralinear region is for 4 synaptic inputs, instead of 5.' This is very confusing, I believe the first comma is misplaced. Regardless, please consider rewording.

Corrected (lines 1119-1121) - thank you.

* Line 1277: Poirazi et al. 2013 -> 2003

Corrected - thank you.

Reviewer 2:

The manuscript of Saray et. al. describes a rigorous and impressive platform, HippoUnit, for the testing of neuron models in a quantitative fashion against a uniform set of experimental data. The technical accomplishment alone is quite impressive, as neuron models are often coded in such distinct fashions that creating a platform that can be readily applied to a wide range of such models is a significant challenge. Tools to perform such tests in a widely applicable manner are, indeed, much needed. On this alone, this manuscript merits strong consideration for publication at *PLoS Computational Biology*.

We thank Reviewer #2 for his/her positive assessment of our work, and especially for the constructive comments that allowed us to further improve our manuscript.

In particular, we decided to follow the advice of the Reviewer and the Editors, and converted the present manuscript into a Methods article, focusing on the description of our software and demonstrating its use, but leaving the investigation of specific scientific questions and philosophical issues to future papers.

In view of this new format and revised general argument, we believe that some of the issues listed below may not be directly relevant to the current manuscript any more. We will state this explicitly for the points affected, and we will be happy to address these issues in more detail if this is still considered necessary.

While I am extremely impressed by the platform (although there are issues with it that need to be addressed, discussed as “major comments” below), I am concerned about the way it is presented by the manuscript. The authors assert throughout that there is a need for “community models” and models that account for a large number of diverse neural dynamics. As currently written, the text seems to argue that this is a clear consensus within the computational neuroscience community and that the inability of models to meet these standards is a clear failing, in turn motivating the tool presented in the manuscript. However, this question is still very much open for debate: many computational neuroscientists have highlighted how “cell-to-cell variability” (see Golowash et. al. *Journal of Neurophysiology* 2002 and Ransdell et. al. *JNeurosci* 2013) necessitates the creation of a multitude of neuron models even from the same population (see Marder and Taylor *Nature Neuroscience* 2011 and Marder and Goaillard *Nature Reviews Neuroscience* 2006, as well as the numerous citations in both pieces on the inherent biological variability in neurons), while others have questioned whether a single “realistic” neuron model is even feasible (see Almog and Korngreen *Journal of* *Neurophysiology* 2016). This underlies my primary concern with the manuscript: in the context of the “debate” outlined above, the manuscript currently lacks a sufficient argument as to whether the testing of multiple neuron models against an external set of experimental data, yielding a quantitative “score” of its validity, is justified and necessary for the field.

Thus, I feel significant work is required to properly contextualize this manuscript within this ongoing debate in the field, more clearly articulate the “problem” that this platform solves, and craft an argument as to why that problem is a significant concern to the community (and in turn how, in solving it, this manuscript moves the field significantly forward). Independent of my personal opinion on this debate, I remain entirely open to publication of this work as long as a reasonable and clear argument is made on this topic, including an acknowledgement of the alternative opinion (and likely a discussion of how this tool will be useful to those who may not agree with the premise that the search for more “uniform” models is necessary).

We thank the Reviewer for pointing out this bias in our original argument. As mentioned above, we have converted our manuscript into a Methods paper. In the revised manuscript, we attempted to avoid taking a strong stand on the relative merits of different modeling strategies. We have removed all references to consensus or community models from the Abstract and the Introduction, and tried to motivate the necessity of developing test suites from a broader perspective. The fact that HippoUnit is expected to be useful also from the perspective of developing community models (which has been argued to be useful and even necessary by some researchers) is described briefly in the “Applications of the HippoUnit test suite” section of the Discussion (lines 1361-1369).

Addressing the following concerns would likely prove useful in crafting this aspect of a revised manuscript:

• How would the pursuit of a “community model” fit with the known “cell-to-cell variability” as described in the articles (included above) by Marder, and her (and others’) conclusion that this necessitates the creation of multiple models to reflect this diversity?

Although this question may not be critical for the revised “Methods” paper, we note that the apparent contradiction between consensus (or community) models and cell-to-cell variability can be resolved if, instead of developing a single “typical” model neuron, the aim is modified to creating a whole population of model neurons with the experimentally observed variability (and average behavior). In fact, we believe that HippoUnit can also be useful in this context, which is now described in some detail in the last paragraph of the new section “Interpreting the results of HippoUnit” in the Discussion (lines 1412-1423).

• Why is the fact that there are “a large number of different models of the same cell type… that were developed for different purposes” necessarily a negative, as is implied in the text at Line 80?

As we converted our manuscript to a Methods paper, we decided not to address this issue in the current article, and removed this argument from the Introduction.

Put alternatively, on Line 690 the authors acknowledge that “the behavior of the different models is very diverse”. Is this a “feature” of the actual biological system, or a “bug” in the current modeling state of the art? It seems that the paper is arguing the later, although many (see the citations included here) would argue the former, or that this diversity is simply a necessity given the current state of the art.

Although this question may not be critical for the revised “Methods” paper, we note that whether the diverse behavior of the existing models should be considered a “bug” or a “feature” depends on whether they collectively display the correct (experimentally observed) kind of diversity. In other words, the question is whether the different existing models could correspond to different members of the real neuronal population. If, instead, we find that each existing model behaves correctly (within the experimentally observed variability) in some domains, but shows behavior that is unlike that of any real neuron in other domains, then this is certainly not a “feature” of existing models – although, as you point out, it still may be “a necessity given the current state of the art.”

• The analogy to software development (Line 87) is an interesting one but lacks sufficient justification for its application here. Why is this analogy appropriate?

Although this analogy is not so prominent in the revised version of the paper (it was removed from the Introduction, although it is mentioned briefly in the Discussion (lines 1366-1369), we think it is appropriate in certain modeling scenarios. In particular, if the goal is develop models that match experimental data in several distinct domains, then it is important to validate the model with respect to all of the relevant criteria after every model iteration, which is similar in spirit to the idea of unit testing in software development.

• On Line 92 the authors admit that the pursuit of “community models… has rarely been attempted in neuroscience.” This begs the question of *why* this is the case. If this pursuit has rarely been attempted in the decades that neuron models have been in use, might there be justification for this? If so, what would be the counterargument to this justification that motivates the pursuit outlined in this paper?

We believe that this point no longer needs to be addressed by the revised “Methods” article.

• The choice of the “features” used to judge the models, as well as the experimental data to compare to, seems in itself inherently subjective, and counter to the idea that HippoUnit provides an “objective” measure of a model’s accuracy. Why were these specific features, and the corresponding experimental data, chosen? Why are these features enough to capture the entirety of a neuron’s dynamics?

Two basic considerations drove the selection of tests for implementation in HippoUnit. First, we tried to include tests that characterize different aspects of neuronal electrophysiology, and that collectively provide a strong set of constraints on the input-output function of CA1 pyramidal cells. However, we do not believe that the current set of tests completely characterizes the physiology of these neurons; for instance, it would be useful to expand HippoUnit by adding new tests on the signal integration of the basal or the more distal apical dendrites. Second, we were severely constrained by the availability of experimental data required for precise, quantitative comparison. We had to find papers that describe the experimental and analysis protocol in enough detail to allow its exact replication in the simulations, and that provide enough information (in the text, tables, or sometimes figures) to compute the experimental mean and standard deviation for the relevant features. We note that we will continue to extend HippoUnit after the publication of this paper, and hope to receive contributions from other members of the community.

We discuss this point in the revised manuscript at lines 569-575 and 1458-1465.

• What about the impact of neural morphology? The authors themselves discuss the variety of morphologies amongst the models that they test, and it is well established that this variability impacts neural dynamics (see Mohan et. al. *Cerebral Cortex* 2015 and Eyal et. al. *eLife* 2016 as just two examples). Is it appropriate to compare dynamics from different neurons, that necessarily have different morphologies?

We think that this question is closely related to the general issue of neuronal variability that we now discuss at some length in the section of the Discussion titled “Interpreting the results of HippoUnit” (lines 1398-1411). The main point is that the tests of HippoUnit take into account most types of variability between neurons (including morphology and biophysical parameters) by considering the variability of the experimentally measured features as well as their means. All of the target experimental features used in the tests of HippoUnit are the mean of features extracted from recorded voltage traces of different cells (of the same cell type) with different morphologies, and the standard deviation is also provided and is taken into account when computing the feature scores. The impact of testing models with different morphologies is then expected to be similar to having cells with different morphologies in the experimental population. In addition, the models tested all had biophysical parameters which had been tuned to reproduce physiological features, and this is expected to compensate (at least partially) for the morphological differences.

While I feel addressing these concerns is necessary as the paper is currently constructed, I acknowledge that this would veer somewhat into the realm of a “philosophical” argument (see Almog and Korngreen) that may not be appropriate or reasonable in a “Research Article”. However, a straightforward solution would be for this piece to be reworked as a “Methods” article. As I’ve mentioned above, the technical prowess required to implement HippoUnit is impressive, and the authors have done a good job outlining multiple fashions in which this tool can be used effectively. Rather than making an argument about the necessity of “model validation” and “community models” that this software may not be equipped to fully answer, a simple presentation of the unique tools provided by this platform would facilitate the use of this tool by computational neuroscientists regardless of their opinion on the “debate” I’ve described here in detail. There are already many portions of the paper that read like a “Methods” article, so I feel that making this change would not be an onerous task.

Regardless of whether the authors choose to address these concerns via a more significant revision and resubmission as a “Research Article”, or by reframing the manuscript as a “Methods” piece, I remain open to reevaluating a piece that addresses the concerns I’ve outlined (and quite interested and excited to see how the authors choose to address these questions). In summary, while HippoUnit itself is impressive and should be of great use to the computational neuroscience community, I feel the manuscript as written may be “overreaching”, and in so doing requiring that the piece answers “philosophical” questions that this tool may not be suited for. However, I believe that with some alteration to its presentation, the manuscript could “describe outstanding methods of exceptional importance that have been shown, or have the promise to provide new biological insights” (taken from the “Scope” for a Methods article), and thus merits strong consideration under the Methods banner.

We agree with the Reviewer that, in its previous form, the manuscript may have been “overreaching”. The main goal of this paper is certainly not to argue about the need of a community model (which we agree is controversial), but to show the utility of HippoUnit (which we believe is relevant in many different contexts). Therefore, we decided to change our manuscript into a Methods article. In this new version of the manuscript the Abstract, the Introduction and most parts of the Discussion were significantly revised to emphasize the motivation behind the development of HippoUnit, and the fields and purposes for which it is advantageous to use in computational neuroscience, while avoiding any strong arguments on the need of community models. We also added a section to the Discussion on how the results of HippoUnit should be interpreted (lines 1377-1423).

Some additional comments, both major and minor, that should be addressed in revisions are included below:

**Major:**

1. As an important example of the issues caused by the lack of proper contextualization, consider the following sentence beginning on line 56: “Applying our tests to several models available in the literature, we show that each model is able to capture some of the important properties of the real neuron but performs badly in other domains.” A similar statement is made on Line 743: “From this figure it is even more clearly visible that each model fits some experimental features well but does not capture others.” This is not necessarily a surprising or novel finding. While to some this reveals the limitations of computational modeling, others see this simply as the reality of the field given the current “state-of-the-art” (the arguments of Almog and Korngreen are illustrative of this perspective). Addressing this concern in the overall context of the current state of neuronal modeling would be an ideal place to start in crafting the “argument” for “community models” that I think is necessary in this piece.

While turning the manuscript into a Methods paper, we thoroughly revised the argument, and tried to avoid statements like the ones pointed out here.

2. The last line of the abstract, stating that this framework has “the aim of facilitating more reproducible and transparent community model building”, is a fantastic statement of the utility of this tool. I feel that this could serve as an extremely useful “thesis statement” for a Methods type piece, as it completely sidesteps the debate about the need for “community models”. If the authors choose to keep this as a Research Article, this would need to be expanded upon, as more concrete examples of how this has been accomplished using this tool would be necessary.

3. A discussion of “cell-to-cell variability” (see Golowash and the articles by Marder) must be included as a “counterpoint” to the second paragraph of the introduction beginning on Line 74. Some neuroscientists would argue it is inappropriate to constrain or compare a neuron model with data from other cells, let alone from other animals and other experimental conditions. This issue arises throughout the paper, as the platform compares cell models to results taken from other literature, and necessarily other cells/animals/experimental protocols. This needs to be directly and thoroughly addressed. This issue comes up again in the Results at Line 766, amongst other places.

We think that there are two separate aspects of this issue that are both considered in the revised manuscript. First, as we already discussed, the variability of cells of a certain type within the same kind of experiment (using the same type of animal, preparation, protocol etc.) is captured by HippoUnit by considering the standard deviation of the experimental data in addition to their mean, which makes it possible to decide whether the behavior of the model is within the normal experimental range. This is described in the new version of the manuscript (lines 595-598 in Results and lines 1398-1411 in the Discussion). On the other hand, variability among different types of experiment, which may be due to differences in animal strain, age, experimental conditions, or recording protocol, cannot be properly captured by the tests of HippoUnit, all of which are based on data from one specific experimental setup. Therefore, when interpreting the results of a test, one should also consider the experimental conditions, and if a model aims to capture neuronal behavior under different conditions, the tests themselves may need to be adapted accordingly. This issue is now also described in the Discussion (lines 1379-1397).

4. Evaluating models based on a “common standardized criteria” (Line 97) might be useful, but the argument for this needs to be more fleshed out. Furthermore, determining *what* these criteria are itself is a complicated and potentially contentious endeavor, so how these criteria were chosen by the authors needs to be more thoroughly developed.

Several (non-exclusive) motivations for using HippoUnit to evaluate models are now described in the Introduction and Discussion (“Applications of the HippoUnit test suite”). The criteria we used to select tests for implementation were described above, and also in the Results section of the manuscript (lines 569-575). Further related thoughts are described in the Discussion under “Extensibility of HippoUnit” (lines 1458-1465).

5. I love the sentence beginning on Line 118: “The test suite that we have developed allows for the quantitative comparison of the behavior of anatomically and biophysically detailed models of CA1 pyramidal neurons with experimental data in all of these domains.” This feels like another part of an ideal “thesis statement” for a Methods piece: instead of having to make the argument for the “significance” of the results obtained using this tool, the authors instead need only describe its power and utility.

6. The way HippoUnit is used in the *development* of new models seems to be one of the most exciting features of this tool, yet it is treated almost as an “afterthought” in the manuscript (it isn’t even mentioned in the introduction until Line 123, the final paragraph). Were this paper to be recontextualized as a Methods article, more strongly emphasizing this application of HippoUnit may significantly benefit the piece.

In the revised manuscript, this topic gets more emphasis in the Abstract (lines 33-35), the Introduction, and the Discussion. As an example of this application the results of the models developed in the Human Brain Project are shown. We also regularly use HippoUnit in our group during the development of models of hippocampal pyramidal cells and also interneurons. As we would like to publish these results in separate papers, and also because these scientific investigations are outside the scope of this “Methods” article, we did not include details on this in the present manuscript.

7. The paragraph beginning on Line 207 is a bit concerning. The statement that the voltage responses “can strongly depend on the experimental method” is true, and arguably could be seen as a flaw in the methodology of HippoUnit. Addressing this by saying the features were “extracted from two different datasets” does not seem sufficient to address this concern, especially considering the known biological variability between similarly classified cells (again, see the various citations above). Similarly, the argument about how this data was determined to be “typical” needs expansion. Many would argue that comparing models to “typical” values is itself a problematic methodology given cell-to-cell variability (see the various citations above); thus, this potential criticism needs to be directly addressed and the choices made in the implementation of HippoUnit more fully justified.

The issue of dependence on experimental conditions is an important one, and we addressed this in the Discussion section of the manuscript (lines 1379-1397; please also refer to our response to point 3 above). The issue of cell-to-cell variability is also discussed extensively in the Discussion (lines 1398-1411; please also refer to several of our responses above). Furthermore, we note that when we talk about “typical” data, we do not simply mean a single average value, but this is meant to include data within the typical experimental range (as characterized, for simplicity, by the mean and the standard deviation of the experimental features). For the specific case of our patch clamp dataset, we said that these data were “typical” because the extracted features were found to be similar to the values found by others in the literature. Therefore, we believe that the recordings are free from significant methodological or other biases, and therefore can be used as representative data. Finally, we suggest that the choice of the target dataset should depend on the goal of the modeling study. In this specific case the models from the literature were compared more extensively to data from patch clamp recordings because all of these models were originally tuned to fit patch clamp experimental results. Comparison of their somatic behavior to the sharp electrode data was performed to see whether they actually fit the patch clamp data better, and this expectation was mostly confirmed by our analysis.

8. HippoUnit is described as being applicable generally to a wide variety of neuron models, implemented in a variety of fashions: the authors assert in the Results that (Line 591) “HippoUnit can be run on neural models that are built in the NEURON simulator software, without any further coding required from the user.” However, in the Methods (Line 449) the authors state that in order to test one of the models described in the paper, an extra modification had to be made to synapse functions. Why is this not evidence that HippoUnit is not as “generalizable” as advertised? Why is this not included in the HippoUnit code proper, but only in a secondary location?	The paragraph in the discussion beginning on Line 1330, in which it is revealed that significant additional work was required to implement the models studied in this work in HippoUnit, also seems contradictory with this, and thus needs to be disclosed more clearly and conspicuously.

Although we made a lot of effort to make HippoUnit as generally applicable as possible, it is impossible to automatically deal with every possible model type and structure that can be implemented in Neuron (within a single script). Modern, standardized model implementations would likely help with this issue (see “Uniform model formats reduce the costs of validation” in the Discussion).

The Poirazi et al. 2003 model is an old one, with a pointer-based activation in their synapse model. This type of synaptic model is not generally used anymore, so we felt it unnecessary to include the code dealing with this in the HippoUnit base code. However, sharing the child class implemented for this purpose gives a good example for users on how one can modify the capability methods of HippoUnit to match their own models or purposes, which is why this code is included in the repository that contains examples of HippoUnit usage. (This point is included in the revised manuscript in lines 462-466)

The need for a “standalone” version of the tested models is discussed several times in the manuscript (lines 547-559, 580-586, 1427-1442). We do not think it is possible to automatically detect and remove all the code in the model files that perform runtime modification, automatic simulations or create built-in graphical user interface. Therefore, this task needs to be carried out manually for most published models, unless the separation of the model itself from additional code was done cleanly in the original implementation.

9. The issue of comparing different neurons with different morphologies (described above) should be discussed in more detail regardless of whether this piece remains a “Research Article” or becomes a “Methods” article.

The issue of different sources of cell-to-cell variability (including morphology), and how HippoUnit deals with this problem, are now described in detail in the Discussion (lines 595-598 and lines 1398-1411 in the revised manuscript; please also refer to our responses above).

10. The issue described in the paragraph beginning on Line 776, in particular that “the models are not compared to exactly the same set of features in the two cases” and that this influences the error scores, could be interpreted as a flaw in HippoUnit that could be addressed by further development of the underlying platform. If it is not, some elaboration on this issue would be useful in assuaging any further criticisms, as the solutions described in the following paragraph seem “ad hoc” at the moment.

We do not think that this issue stems from any particular flaw in HippoUnit or eFEL (the feature extraction package used in the Somatic Feature Test). The root of the problem that we identified is that the two datasets were recorded not only using different recording methods – patch clamp and sharp electrode – but (partly as a consequence) also using different protocols (current amplitudes, current duration etc.), and therefore provide different sets of features. As an important example, voltage traces with and without action potentials clearly provide different types of features. Also note that the same electrophysiological parameter can often be extracted from multiple voltage traces, and these are all treated as separate features in the test, so a difference in the number of recorded traces automatically leads to a difference in the set of features. These issues make it more difficult to compare test results obtained from different datasets; however, by identifying the exact sources of potential bias, we were able to improve the comparison using the approach described in the paper.

This issue is discussed in the revised manuscript at lines 805-852.

11. On Line 845 the authors discuss how three models were able to “cheat” the Depolarization Block Test. This could be read as undermining the overall efficacy of HippoUnit, especially given its stated goal to be applicable to a wide variety of models. At minimum, an explanation as to why further steps weren’t taken to adjust the platform for these “cheats” is necessary (a statement that this would be computationally problematic/inefficient/etc. would be completely reasonable if this was the case), as well as perhaps further discussion as to how one might deal with similar “cheats” when this platform is applied to other models.

The way in which certain models may be able to “cheat” the test is certainly relevant for the applicability of HippoUnit, and we have added a paragraph discussing this issue to the section “Depolarization Block Test” in Results. The main point is that the behavior of these models is so similar to depolarization block that it is hard to distinguish them using automated methods, although a trained researcher can tell them apart by looking at the voltage traces and other outputs of the test. We were also concerned that adding further (ad hoc) modifications to the test so that it produces the correct output in these particular cases might lead to incorrect results in future test cases. The general conclusion is that models (especially when they are not at all tuned to show the tested behavior) may exhibit a wide variety of responses, and it may not be possible to develop a test that can perfectly deal with all the potentially occurring, otherwise unexpectable behaviors. On the other hand, the output of HippoUnit (beyond the final score), and especially the figures of the recorded voltage traces, make it possible for the user to identify whether the model ‘cheated’ the test, and revise the conclusions accordingly. (These points are discussed in the revised manuscript in lines 913-921.) Finally, we will be happy to further develop HippoUnit to make it more efficient or more general, and by making it open source we made it possible also for others to improve it, or share ideas (e.g., in form of Issues on GitHub).

12. The fact that the Golding et. al. models do not perform well on the Back-Propagating Action Potential Test, despite the fact that they were developed to account for this feature (Line 1109), is critical. If this model was designed to exhibit this behavior, what does that say about the test implemented in HippoUnit?

One interpretation might be that this shows why it might be inappropriate to compare models to experimental data obtained from different neurons in a different experimental setting. Something similar appears to be the case for the Gomez Gonzalez et. al. models and the Oblique Integration Test. Both these points need to be thoroughly addressed.

The tests of HippoUnit were designed to mimic the experimental protocol used to gather the target data as precisely as possible, in order to make the comparison between the models’ behavior and the experimental data fair and complete. As it turns out, this approach was not followed so strictly in Golding et al. (2001), where they used different protocols and evaluation methods for the experimental data and the models.

More specifically, in the models they evoked only a single action potential (not an action potential train) and compared the AP amplitude at the soma and at a single distance on the apical trunk (375 µm) (Fig 8 of Golding et al. 2001). Furthermore, they only made a visual, qualitative comparison, showing the recorded voltage traces. They also added a color-coded morphology to show the voltage changes due to the somatic current injection in the different models (Fig 8 of Golding et al. 2001), but it lacks a scale bar, which makes it difficult to make the comparison about how far the AP invades the dendrites, to the experimental results. By contrast, the bAP test of HippoUnit attempts to replicate the experimental conditions used by Golding et al. more precisely (by evoking AP trains), and takes into account more features (including the amplitudes of the first and last action potentials at several different distances) for the comparison.

In summary, models in the Golding et al. 2001 paper were not tested as extensively, and their behavior was not compared quantitatively to their experimental data, the way that HippoUnit does. Therefore, the output of HippoUnit reveals differences between their model’s behavior and their experimental data, in domains in which their models were not tested in the original publication.

The issue is discussed in the revised manuscript in lines 1162-1174.

In the case of the Gómez González models and the Oblique Integration test, it can again be said that HippoUnit tests the models more extensively than they did in the paper, as HippoUnit selects and tests all the relevant oblique dendrites according to the experimental protocol.

In addition, as described in the manuscript, it seems that in the Gómez González paper they selected oblique dendrites by measuring Euclidean distance from the soma, while we find it more appropriate to measure the path distance (along the dendrites), and therefore HippoUnit is implemented this way. This results in the selection of a partially different set of oblique dendrites from those studied and adjusted in the Gómez González paper. When running the Oblique Integration test on these models, the output of HippoUnit shows that the different oblique dendrites of these models can show quite diverse behavior, and beyond those studied in the corresponding paper, other oblique dendrites do not necessarily match the experimental observations (lines 1189-1192 in the revised manuscript).

13. The opening sentence of the paragraph beginning on Line 1303 is very well put. However, it seems out of place relative to the rest of the paper, which seems (intentionally or unintentionally) to advocate that models that aren’t validated in a wide range of settings aren’t “good enough”. I think the rest of the paper could benefit from keeping the tone/perspective from this section (which may be facilitated by the transition to a “Methods” article). In particular, stating that “no single model should be expected to achieve an arbitrarily low score on all of the validation tests” (Line 1318) is an important statement that needs to be made more prominently in the manuscript.

In converting our manuscript into a Methods article, we shifted the emphasis closer to what is suggested here, and hopefully eliminated controversial, unsupported statements.

**Minor:**

1. On Line 21 the authors state that neuronal models “can be” useful. This seems unnecessarily deferential, especially considering that it is argued how important they are throughout the rest of the paper.

This part has been rewritten in the revised manuscript.

2. The term “validation” is used throughout the paper, but this is a term that can be interpreted in a variety of different ways. I would avoid using this term in the abstract and title if at all possible, and then introduce how this term is being used in this manuscript thoroughly in the introduction. “Accurate” is another such term that might benefit from expounding upon.

The title and the abstract have been changed, and the term “validation” is now better described in the revised Introduction.

3. On Line 179, the assertion that HippoUnit’s scores “better fit its tests and the observations belonging to them” needs to be elaborated on further… “better” is an inherently subjective term and requires some sort of argument justifying its use.

The revised manuscript contains a more complete description of why and how the scores of HippoUnit differ from those of SciUnit and NeuronUnit at lines 187-194.

4. The fact that the AP amplitude has such a broad range (Line 236) could be interpreted as a strong piece of evidence for a “counter-argument” against the methodology taken in designing HIppoUnit. A stronger argument as to why this is acceptable in the design of this tool is necessary, especially given the context of the “community model” debate.

The most likely reason for the broad range of AP amplitudes is differences in experimental conditions. It is clear that it is impossible to model all of these experimental data using a single model. This means that the modeler needs to specify not only what type of cell they want to model, but also under what circumstances and using what protocol, and should choose the target experimental data accordingly. We think this also applies to community models – but this is not the focus of the current paper. This is why we designed HippoUnit in a way that it is relatively simple to change the target features if the necessary experimental data are available. These points are also included in the Discussion of the paper.

5. The tool for classifying the apical sections of pyramidal cells (Line 485) is very impressive, and in itself extremely useful as a uniform mechanism for classifying sections in a NEURON model. Might this be more strongly highlighted (especially if the article becomes a “Methods” piece)?

This tool relies heavily on the work of others, which is indicated in the script and now in the Acknowledgements as well. Therefore, we decided to describe mainly those improvements that were developed in the context of the current study.

6. In the “Models from literature” section of the Methods (Line 521), each model is described in detail, including what the model was initially designed for. I feel that this knowledge is crucial in order to evaluate the Results, and thus might be more appropriate as the first section of the Results. If the authors choose not to make this change, a brief “refresher” on this topic would be useful to begin the Results.

We agree and moved the description of the models to the Results section (lines 635-659).

7. The sentence beginning on Line 568 regarding the modeler’s feedback seems superfluous, especially since feedback was received from only two of the groups. I would suggest eliminating this sentence, or if not further expanding upon what this feedback contributed to the work.

We agree and deleted that part.

8. The opening sentence of the Results (beginning on Line 577) already reads more like a “Methods” paper than a “Research Article” (and is extremely well written!), and might facilitate that transition if the authors choose.

9. The argument made in the paragraph beginning on Line 661 is already made in the Methods (i.e. that the data used in HippoUnit matches the available literature), and probably doesn’t need to be made in full again.

We agree and made that part more concise.

10. It may be illustrative for the authors to compare their Figure 3 to some of the figures in the two Marder papers cited here. In those papers, very similar types of figures are used to argue that biological variability exists in neurons and necessarily should influence the creation of neuron models, whereas here the authors seem to use this figure as evidence that the existing neuron models are lacking. Reconciling this could prove useful in crafting the proper context for this manuscript.

Figure 3 shows not only the variability of the models, but also includes, in addition to the experimental mean, a representation of the experimentally observed variability (standard deviation). This makes it possible to tell whether (and by how much) a certain feature extracted from a model is outside the experimental variability. The correct interpretation of the results of HippoUnit is now discussed in detail in the Discussion (lines 1379-1423).

11. If “not all the observation features can be evaluated for each of the models” (Line 819), is it appropriate to include those features in HippoUnit? Why not eliminate them and only compare “apples to apples”, i.e. features that can be tested in all of the models? This should be addressed in more detail.

The most common reason why a particular feature cannot be evaluated for a given model in a specific protocol is that the model behaves qualitatively differently from the experimental data under those conditions. A typical example is that spike features cannot be extracted from the voltage trace when no action potentials are evoked by that particular current injection. Therefore, the fact that a particular feature could not be evaluated for some models typically does not reflect any bug or deficiency in HippoUnit (or the feature extraction package eFEL). In this particular case, the fact that a very different number of features could be evaluated for the models is an indication that some models match the data better than others even at the qualitative level. In addition, for the comparison between the two datasets, we already removed those eFEL features that could not be evaluated in one of the datasets; also removing all of the features that could not be evaluated for one of the models (using the protocol corresponding to at least one dataset) would have left us with a drastically reduced feature set that probably would not represent all aspects of the somatic responses (for instance, spiking features could be completely absent). This is addressed in the revised manuscript at lines 811-820.

12. I would suggest reworking the opening paragraph of the Discussion (beginning on Line 1264). Many of the statements in this paragraph are opinions (albeit well supported ones), but as currently written they come across as statement of fact. Some more citations and specific examples backing up these statements would likely help.

The discussion has been completely reworked.

13. A mention of the Neurodata Without Borders initiative seems wanting in the paragraph beginning on Line 1346.

The paragraph in question is about how standardized descriptions of models are expected to aid automated testing. Although there are now a number of initiatives with the goal of creating standardized representations of data in neuroscience, we are not aware of any others beyond those already listed that specifically aim to handle models. We have looked at the information we could find on the website of the Neurodata Without Borders initiative, but we could not find any indication that it was also targeting models. However, if this is considered critical, we will be happy to discuss this and other initiatives that do not specifically target models.

Note related to data access:

As PLOS data access policy does not allow notes like “data not shown”, we provided the accessibility of the mentioned data in lines 964-966 and 729-731, and deleted another non-essential reference from the sentence starting at line 757.
