# Supplementary material for: HippoUnit: A software tool for the automated testing and systematic comparison of detailed models of hippocampal neurons based on electrophysiological data
Source: PLoS Comput Biol. 2021 Jan 29;17(1):e1008114. doi: 10.1371/journal.pcbi.1008114 (PMC7875359; doi:10.1371/journal.pcbi.1008114)
Supplement: Attachment — Submitted filename: response_to_reviewers_Sarayetal2020_20201210.docx [file pcbi.1008114.s004.docx]

Dear Prof. Lytton and Prof. Blackwell,

We sincerely thank you and both reviewers for dedicating the time and effort to reviewing our substantially revised manuscript, and for the invitation to submit a revised version again. We are pleased to see that both reviewers appreciated our revised manuscript that has been reframed as a Methods paper. We are grateful for the additional insightful comments and suggestions of Reviewer #2. We have considered them carefully and addressed them both in our revised manuscript and in our responses below. In the uploaded "Revised Article with Changes Highlighted" file we marked the changes of the manuscript in yellow.

We hope that our revised submission will be received favorably at the journal, and look forward to hearing from you.

Yours sincerely,

Sára Sáray

Reviewer #2: The authors have performed considerable work to reframe this manuscript as a Methods piece, which I think has improved it dramatically, and I applaud them for putting in this effort. As the authors mention, many of my initial comments on the manuscript have been resolved through this change. At this point, I am ready to endorse publication of this manuscript following the consideration of the following comments, which I think should be easily doable especially relative to the first-stage revisions.

Thank you. We have considered all your comments, and revised the manuscript accordingly.

1) The largest issue I had with the revised manuscript was confusion regarding the use of patch clamp versus sharp electrode data. The discussion of this at the beginning of the "Somatic Features Test" section (Lines 685-689) seems to make the argument that the patch clamp data is more appropriate in the setting of the explorations performed in the manuscript. However, beginning around Line 800 the sharp electrode data is reintroduced without much explanation, and it's said that both datasets were used. Then, at Line 821 the authors state that initial explorations show that most models actually fit the sharp electrode recordings better, which confuses things further. Only after some "recalculations" (Line 851) is it concluded that there is a superior fit to the patch clamp data.

Personally, I feel that this needlessly confounds the overall story of the "Somatic Features Test" section, and I'm not sure that the sharp electrode recordings add something "necessary" to the piece (although I acknowledge that, given my confusion interpreting this portion of the manuscript, I may have overlooked other parts of the paper where the sharp electrode data is more necessary). I would ask the authors to review this aspect of the work in detail, and ask themselves three questions: first, whether both data sets are necessary for the totality of the manuscript; second, whether both data sets are necessary in the "Somatic Features Test" section specifically; and third, depending on the conclusions reached for the first two questions, how the necessity of both data sets can be put forth in a more clear and succinct manner.

We agree that the comparison of the models from literature to both datasets in the "Somatic Features Test" section is not essential for the basic message of the manuscript. We also admit that, mainly due to the necessity of discussing various biasing factors in this comparison, this section may have been difficult to digest and possibly confusing. Therefore, we decided to delete this part (along with the associated figure), and we evaluate the models from literature only against the patch clamp dataset in the Results section. Nevertheless, as we thought that this use case might be interesting for some readers, we added a paragraph that briefly discusses this possibility to the “Applications of the HippoUnit test suite” section in the Discussion (lines 1299-1308).

On the other hand, as the HBP models were optimized using sharp electrode data, the sharp electrode dataset is still described in the manuscript and is used in the “Application of HippoUnit to models built using automated parameter optimization within the Human Brain Project “ section.

2) In their response, the authors note that "models in the Golding et al. 2001 paper were not tested as extensively" as others in the manuscript. I wonder if that might unnecessarily confound the comparisons throughout the paper then. Might the paper read more clearly if the Golding models were simply eliminated? This might help to make the paper more digestible overall, as it is already quite voluminous and dense. I leave this choice to the authors, however.

We believe that this comment stems from a misunderstanding of our earlier response. We tested all of the models from the literature, including the Golding et al. model, in exactly the same way. What we meant to highlight in our response was that the authors of the Golding et al. 2001 paper did not test their models as extensively as we now did using HippoUnit. As we described the issue in the manuscript: “…in the original study [i.e., the Golding et al. 2001 paper], only a few features of the model’s response were compared with the experimental results. HippoUnit tested the behavior of the model based on a larger set of experimental features from the original study, and was therefore able to uncover differences between the model’s response and the experimental data on features for which the model was not evaluated in the source publication.”

3) I find myself a bit concerned by the author's response to my comment 11, regarding the fact that "some features cannot be evaluated for a given model [because] the model behaves qualitatively differently from the experimental data". While the authors have indeed discussed this in the revised manuscript as mentioned, I believe that this merits a much more detailed discussion in light of the quoted comment above. I could be misunderstanding the author's response, but while it's true that if a model does not exhibit action potentials under a certain protocol spiking features can't be evaluated, if the experimental data exhibits spiking but the model doesn't, this is a major issue. Essentially, I find myself more confused by where HippoUnit stands on this issue based on the author's response to my comment than I was previously. While I don't think this is a "dealbreaker" by any means, I would appreciate at minimum for this to be expounded upon in a response, and ideally for there to be some furtherdetailed discussion of these issues in the text.

We agree that it can be considered a major (biological) difference between the model’s behavior and the experimental data if, for a given input current, the model does not exhibit action potentials while the experimentally recorded neuron does (or vice versa). This also presents a technical issue in the comparison as spike features can be extracted from one of the responses but not from the other. Therefore, this is a known issue that we had to deal with when we designed our tests.

One possible solution that we considered was using a penalty as the feature score each time a spiking (or other) feature cannot be evaluated for the model. We decided not to use this solution as it is rather arbitrary and raises further issues, such as how large the penalties should be, or whether we should apply the same penalty for each unevaluated feature or perhaps larger for those thought to be more important. The answer to these questions, in our opinion, depends on the goal of the user. For example, it may not be a critical problem if a given model starts to fire only for a slightly larger current amplitude than observed experimentally. This is especially true since action potentials near threshold are often noise-driven, but the noise that is present in the experimental data is typically omitted from the models. Overall, adding arbitrary penalties to the scores could easily mislead the user about the performance of a model.

Therefore, we decided to deal with this issue in a different way. On one hand, the feature scores show the discrepancy between the model’s features and the experimental features for those features that could be evaluated for the model. This includes features (such as spike count) that indicate the lack of spiking in the model when there are action potentials in the data. On the other hand, the output of HippoUnit contains the number of successfully evaluated features (as proportion to all the features – also shown in Fig 4 B of the manuscript), and the names of the unevaluated features are also printed out and saved in the “text_log.txt” file. This information helps the user interpret the results and decide how to deal with any possible issues.

Further detailed discussion on how HippoUnit deals with the issue can be found in lines 710- 718 and 791-793 of the revised manuscript.

4) A couple times in the text (for instance, on line 42) the authors make statements along the lines of "each of these models provides a good match to experimental results in some domains but not in others". This is a somewhat obvious assertion, and I do not believe the authors intend it to be the "crux" of the argument of their paper, but the way it is seemingly highlighted places more weight on this quite intuitive assertion than I think the authors intended. I think such a statement is distracting and can be removed (especially since the authors will "show" this to be the case via their use of HippoUnit, and thus don't need to "tell" us explicitly). Indeed, if a would-be reader were to conclude that this were the overall argument of the paper, they might be unintentionally turned off.

Thank you for pointing this out. We agree, and rephrased the affected lines in the Abstract and Author summary (lines 39-43 and 59-61).

5) The authors mention that the cells of interest are rodent neurons somewhat inconsistently. Especially considering the burgeoning literature on neural modeling in humans and non-human primates, I would ask that the authors consistently refer to "rodent hippocampal CA1 pyramidal cells" to avoid inadvertent confusion.

Thank you for pointing this out. As all the experimental data and models used in this study are related to the rat hippocampus, we added the word “rat” (instead of “rodent”) to each mention of the CA1 PC in the manuscript.

6) There are typos on Line 82 (a space is needed between 131 and different), potentially on Line 222 (in the mention of the patch clamp dataset), and on Line 1219 (interneurons, not interneuron, as first word).

Corrected – Thank you for spotting them. (We found no typo in Line 222.)

7) I would eliminate the phrase "common, standardized criteria" on Line 94, which I believe veers too close to the "philosophical" arguments that have been side-stepped by the new Methods format.

We have replaced this phrase with “the same set of well-defined criteria,” which does not imply standardization.

8) While I appreciate the inclusion of the first paragraph in the "Interpreting the results of HippoUnit" section, which goes to some of my initial concerns, I feel like this section (along with perhaps the concluding sentence beginning on Line 919 of the "Depolarization Block Test" section) may read to some like "counter arguments" to the paper itself. It is somewhat jarring to read about the importance of the creation of HippoUnit, who's primary output is scores on validation tests, only to later be told that "a high final score on a validation test using a particular experimental dataset does not mean that the model is not good enough or cannot be useful for a variety of purposes". Ideally, I would like to see this "tone" included consistently throughout the paper, but I recognize that may be a manifestation of my own "philosophical" bias. At this point I do not think that this issue is a "dealbreaker" when it comes to publication, but I would ask that the authors spend some time thinking about how these critical caveats might be made to seem less jarring when they're brought up... this could perhaps be accomplished just by some increased foreshadowing at appropriate points in the text.

We added some thoughts to the manuscript on this at lines 610-616, 715-718, 1353-1357.


While this may seem like a long list, I emphasize that I am enthusiastically endorsing the publication of this manuscript with these comments considered, and I feel like these are relatively minor issues that likely arose given the significant rewrites that the authors performed. It is inevitable that some new issues would arise following such a significant rewrite, and once again I applaud the authors for putting forth the time and effort to do so. With these issues addressed, I feel this will be a more complete Methods manuscript and a strong publication in PLoS Computational Biology.
